# GReat-Child Trial™ based on social cognitive theory improved knowledge, attitudes and practices toward whole grains among Malaysian overweight and obese children

**DOI:** 10.1186/s12889-019-7888-5

**Published:** 2019-11-27

**Authors:** H. C. Koo, B. K. Poh, A. T. Ruzita

**Affiliations:** 1 Nutritional Sciences Programme & Centre for Community Health, Faculty of Health Sciences, Universiti Kebangsaan Malaysia, Jalan Raja Muda Abdul Aziz, 50300 Kuala Lumpur, Malaysia; 2 Department of Bioscience, Faculty of Applied Sciences, Tunku Abdul Rahman University College, Kuala Lumpur, Malaysia

**Keywords:** Attitudes, Intervention, Knowledge, Practice, Children, Whole grains

## Abstract

**Background:**

Studies have reported that improvement of dietary habits through increased whole grain foods consumption at an early age has the potential to lead to betterment in lifelong health and wellness. The GReat-Child Trial™ was a 12-week quasi-experimental study with 6 months follow-up investigating a multi-component whole grain intervention, which consisted of behavioral, personal and environmental factors based on Social Cognitive Theory (SCT). This study aimed to evaluate the feasibility and acceptability of the GReat-Child Trial™, as well as to determine the changes in knowledge, attitudes and practices (KAP) of whole grains consumption among overweight/obese children.

**Methods:**

Two schools in Kuala Lumpur with similar socio-demographic characteristics were assigned as intervention (IG) and control (CG), respectively. Inclusion criteria were healthy Malaysian overweight/obese children aged 9 to 11 years who had no serious co-morbidity. Children who reported consuming whole grain foods in their 3-day diet-recall during recruitment were excluded. A total of 63 children (31 IG; 32 CG) completed the intervention. KAP questionnaire was self-administered at baseline [T0] and post intervention (at 3rd [T1] and 9th month [T2]). The baseline differences between the IG and CG across socio-demographics and scores of KAP toward whole grains were determined using chi-square and t-test, respectively. ANCOVA was performed to determine the effect of the GReat-Child Trial™ on KAP towards whole grains at post-intervention and follow-up. Baseline variables were considered as covariates.

**Results:**

The IG attained significantly higher scores in knowledge (mean difference = 4.23; 95% CI: 3.82, 4.64; *p* < 0.001), attitudes (mean difference = 7.39; 95% CI: 6.36, 8.42; *p* < 0.001) and practice (mean difference = 6.13; 95% CI: 4.49, 7.77; *p* < 0.001) of whole grain consumption compared to the CG, after adjusting for confounders. The IG reported significantly higher scores in knowledge (mean difference = 6.84; 95% CI: 6.53, 7.15; *p* < 0.001), attitudes (mean difference = 9.16; 95% CI: 8.08, 10.24; *p* < 0.001) and practice (mean difference = 8.03; 95% CI: 5.34, 10.73; *p* < 0.001) towards whole grains at T2 compared to T0.

**Conclusions:**

These findings indicate that this intervention made a positive impact on improving children’s KAP on whole grains. We anticipate the GReat-Child Trial™ to be a program that could be incorporated into school interventions to improve whole grain consumption among Malaysian children for obesity prevention.

## Introduction

Grains or rice are the main staple foods consumed by Malaysians. They are universally recognized as the cornerstone of dietary recommendations along-side other carbohydrate-rich sources and constitute the largest component of recommended daily intake in all dietary guidelines [1, 2]. Considering their important role in most diets around the world, interest in the health effects of grain consumption is increasing tremendously, and in particular whole grain [3]. Whole grain consist of germ, bran and starchy endosperm, which are present in the same relative proportions as they exist in the intact grain; in contrast with refined grains, which have had their bran and germ removed during the milling process [3]. The protective effects of whole grain intake have been attributed mostly to the germ and bran components, including fiber, B vitamins and bio-active compounds, such as minerals and antioxidants [3]. Evidence from a recent meta-analysis demonstrated inverse associations of whole grain intake with coronary heart disease, cardiovascular disease, total cancer, diabetes and obesity in adults [4]. There is broad consensus on the health benefits of consuming whole grain in children. Habitual consumption of whole grain foods has positive impact on weight management [5, 6] and lowers serum insulin in children [7]. Considering that the rate of childhood obesity in Malaysia have been rising dramatically, from 5.4% in 2006 [8], to 11.9% in 2015 [9]; greater consumption of whole grain may improve weight management among Malaysian children [6]. The outcome of whole grain consumption in weight management can be the cornerstone for community health activities initiated by government and non-governmental agencies, industries and researchers, to increase whole grain consumption among children, particularly among those who are overweight or obese.

With respect to the health benefits of whole grain, many countries include recommendation to increase intake of whole grain as the main source of carbohydrates in their dietary guidelines [10]. The United States Dietary Guidelines [1] and the Malaysian Dietary Guidelines for Children and Adolescents [2] include recommendations that at least half of all grains consumed should be whole grain. In spite of these recommendations, whole grain consumption varies according to countries and cultures [10]. A longitudinal study from the United States, conducted among the children and adults, found a small increasing trend on whole grain intake, which include breads, ready-to-eat cereals and pastas [11]. However, recent studies revealed that whole grain consumption among children in the United States, Malaysia and other countries are below the dietary guideline recommendation [8, 12–14]. A recent study in Malaysia reported that only 25% of children consumed whole grain, with mean daily intake of 2.3 g/d [13]. Reasons for low whole grain intake are complex but may be due to factors related to lack of knowledge on identification of whole grain, sensory properties especially texture and taste of whole grain foods, lack of varieties and convenient availability [15]. Whole grain products may have a hard crunch or a light and airy texture, and the bitter taste is caused by the phenolic acid and tannins in the bran layer of whole grain [3]. Family preference and food culture are important barriers to whole grain consumption. The majority of Malaysians appear to prefer refined grains to whole grain products [13]. These effects are also likely to negatively impact on intake of whole grain foods consumed during family meals, either at home or out-of-home.

Studies from Malaysia clearly indicate that knowledge, attitudes and practices (KAP) towards whole grains consumption in children were far from satisfactory [16, 17]. Nutrition education intervention which applied Social Cognitive Theory (SCT) and focus on behavior change, has the potential effect of improving knowledge, attitudes and consumption behavior of whole grain foods. Bandura’s SCT is an interpersonal theory that emphasizes mutual interactions of personal, behavior and environment factors [18]. It has been applied extensively in several multi-component interventions involving children that had resulted in positive dietary behavior change [19, 20]. The SCT provides a theoretical foundation for identifying modifiable behaviors, explains human behavior in terms of reciprocal relationships, as well as the triadic and dynamic relationships in which personal characteristics, behavior patterns and environmental influences interact [18]. The SCT constructs were addressed in the GReat-Child Trial™, including behavior factors, such as behavioral capability (whole grain knowledge and behavioral skills) and usual food choice (choice between pairs of foods); personal factors, such as self-regulation (goal-setting), self-efficacy (confidence to carry out behaviors successfully) and outcome expectancies (preferences); and environmental factors consisting of the availability and accessibility of whole grain foods prepared by parents at home.

Development of habitual food choice is multifactorial. Previous research has suggested that good dietary patterns developed at a young age, may lead to healthy eating habits in the future [21]. Therefore, efforts to improve dietary habits through increased consumption of whole grain foods at an early age has the potential to improve lifelong health and wellness [12]. A multi-component school-based pilot intervention utilizing a quasi-experimental study design and consisting of a 5-lesson classroom curriculum based on SCT, which aimed to increase whole grain intake among 4th and 5th grade children, was conducted in the United States; however, this trial did not investigate the effect of the intervention in improving attitudes toward whole grain consumption among children [19]. Thus, the present study (GReat-Child Trial™) was carried out to improve the KAP of whole grains consumption among children with overweight and obesity. The research study protocol that describes the development of the intervention [22], and the effect of GReat-Child Trial™ on weight management have been published elsewhere [6]. The present paper describes the feasibility and acceptability of the GReat-Child Trial™, as well as to investigate the changes on KAP of whole grains consumption among children who were overweight or obese.

## Methodology

### Study design and recruitment of children

The GReat-Child Trial’s overview, rationale, trial design and methods have been described in detail elsewhere (Koo et al. 2016). In brief, the GReat-Child Trial™ was a 12-week non-blinded quasi-experimental intervention that was conducted in March – May 2014, with a 6-month follow-up from June to November 2014. Two schools were recruited from a randomly selected zone in Kuala Lumpur, Malaysia. All the children recruited from the intervention and control schools were respectively assigned to the intervention (IG) and control (CG) groups. Schools with similar socio-demographic characteristics, including household income, educational background and religious affiliation, were assigned as intervention and control on a non-randomized basis. This was to ensure that the IG and the CG were completely independent, with location sufficiently far apart (10 km distance), so they would not have any effect on each other [23]. The inclusion criteria for the subjects were: (1) healthy Malaysian children aged 9–11 years and studying in Primary 4 and 5; (2) children who were overweight or obese; (3) able to read, write and understand Malay; and (4) at least one parent perceived that their child has a weight problem, and was willing to attend individual diet counseling. Children were excluded if: (1) they have a serious co-morbidity requiring treatment, such as diabetes on insulin treatment; (2) on gluten-free diet; and (3) any of their three-day 24-h diet-recall during recruitment indicated that they had consumed whole grain foods.

The SCT concepts and applications employed in the present trial are shown in Table 1. This trial consisted of three components addressing personal, behavioral and environmental factors based on the SCT. The program consisted of: (1) six nutrition education classes were conducted on a fortnightly basis, right after regular school hours and before the children began their after-school co-curricular activities. Each class was 30 min in duration and employed the visual plate model and food guide pyramid to emphasize; (2) 12-week feeding of whole-grain foods (whole-grain biscuits, whole-grain bread and whole-grain ready-to-eat cereal) on a daily basis during recess time at school, to replace any food that the children would normally eat during recess time; and (3) family involvement component where a parent attended an hour of individual diet counselling to practice a balanced diet and to encourage the availability of whole grain foods at home.

**Table 1:** Social Cognitive Theory concepts and application for GReat-Child trial

SCT constructs	Application	Learning activities
Behavioral Domain		
Behavioral capability	Provided whole grain and healthy eating knowledge.	• Participation in whole grain and healthy balanced diet quiz in six times of 30-min nutrition education classes. • Children were required to draw the healthy plate model on the blackboard. • Using food labels to identify foods as whole grain or refined grains. • Locating grain food group and grain food items on the Food Guide Pyramid. • Individual diet counselling for the parents to identify the whole-grain foods, as well as the advantages of whole grain consumption.
Food choice	Served a variety of whole grain foods on a daily basis.	• Tasting whole grain foods for taste, appearance, texture and acceptance. • Researcher introducing variety of whole-grain food during school delivery and let the children familiarizing with it. • Identifying whole grain and healthy balanced diet’s labels during individual diet counselling for the parents.
Personal Domain		
Self-efficacy	Provided practical experiences that emphasized tasting, selecting and preparing whole grain foods.	• Whole grain recipe booklet provided to parents during individual diet counselling, to help them prepare and serve whole-grain food at home. • Handy tips included in the whole grain booklet during individual diet counselling, to show the parents easier way to achieve the recommendation to eat half of the grains as whole grain. • Learning about menu planning for balanced diet during six 30-min nutrition education and individual diet counselling. • Reading food and nutrition labels on the packaging of whole grain foods, to overcome barriers in selecting and identifying the whole-grain foods.
Reinforcements	Children were rewarded and received praise when they correctly answer questions on whole-grain and healthy eating diet.	• Participation in whole grain and healthy balanced diet quiz during nutrition education classes.
Environmental Domain		
Observational learning	Served a variety of whole grain foods on a daily basis.	• Children observed how to prepare a convenient whole grain breakfast. • Whole grain food recipes provided to parents.
Availability	Provided information to encourage the parents to increase availability of whole-grain foods and healthy diet at home.	• Individual diet counselling and booklet provided to advocate for consumption of more whole grain foods, as well as balanced diet at home.

Ethical approval was granted by the Research Ethics Committee of Universiti Kebangsaan Malaysia (Project Code: NN-070-2014). All procedures were conducted in accordance with the guidelines laid down in the Declaration of Helsinki. Permission to carry out data collection was obtained from the Ministry of Education, Malaysia and the Kuala Lumpur Federal Territory Education Department. Permission was also sought from the principals of the selected schools and teachers who were notified about the study. A cluster sampling method was employed to recruit children who fulfilled the inclusion criteria. A minimum sample size of 76 children was calculated using Naing (2009)‘s equation [24], taking into account a non-response rate of 50%. Signed informed consent for this trial was obtained from parents or guardian prior to the baseline data collection. Verbal assent was also obtained from the children prior to the study. Children in IG received a 12-week intervention programme with another 6 months of follow up. In contrast, the children in CG did not receive any intervention, but for ethical purposes, a 2-h general health talk on healthy eating, whole grain choices and physical activity was conducted after the entire GReat-Child Trial™ was completed.

### Data collection and study questionnaire

Outcomes on KAP of whole grains consumption in both IG and CG, were assessed using a questionnaire at three points: baseline (T0); after 12 weeks of intervention, at week 13 (T1); and 6 months after intervention, at 9th month follow-up (T2). The questionnaire was self-administered by the children themselves and supervised by a trained researcher, who provided explanations about any items in the questionnaire when asked. All data collection was done by the same researcher.

KAP of whole grains consumption were obtained using a validated, guided, self-administered questionnaire in the Malay language. The development, validation, establishment of reliability procedures and details of the questionnaire has been described in detail elsewhere [25]. The questionnaire consisted of four parts: (1) sociodemographic characteristics, (2) knowledge domain, (3) attitudes domain and (4) practice domain. The knowledge domain consisted of 15 nutrition and whole grain knowledge-related questions with 2-point scale (0 = wrong or “not sure” response, 1 = correct response), with a total of 15 points. The attitudes domain included 15 items measured attitudes toward whole grain consumption with a total of 75 points, using a 5-point Likert scale for positive items (1 = strongly disagree, 2 = disagree, 3 = neutral, 4 = agree, 5 = strongly agree) and negative items (5 = strongly disagree, 4 = disagree, 3 = neutral, 2 = agree, 1 = strongly agree), accordingly. The practice domain comprised 10 items on whole grain consumption practices. Each question was scored using a 5-point scale (1 = never, 2 = seldom [not in “sometimes” or “always” categories], 3 = sometimes [14 days in a month], 4 = always [4–6 times in a week], 5 = everyday), with a total of 50 points.

### Feasibility and acceptability of the study

Participation and attendance at nutrition education classes were documented to evaluate adherence rates and attrition in the IG and CG. Following the completion of the intervention, a 13-item questionnaire was administered to assess the degree to which children from IG (1) followed through the objectives, (2) gained whole grain-related knowledge and (3) satisfied with the GReat-Child Trial™ module and arrangement. First item was to quantify the degree to which children were able to follow through the overall learning objectives. Second to fourth items were to assess the level of knowledge on whole grain-related that was gained by the children. The last 9 items quantified the degree of satisfaction toward the GReat-Child Trial’s module and arrangement. All statements were scored on 4-point Likert scale ranging from 1 (strongly disagree) to 4 (strongly agree), with higher scores indicated greater feasibility and acceptability.

### Physical characteristics

The method used to access physical characteristics was anthropometric measurements, which had been described in detail elsewhere [6]. In brief, body weight and height were measured twice, according to standard procedures, using a calibrated Tanita digital scale Model SC-330 (Tanita Co., Tokyo, Japan) and SECA Bodymeter 206 (SECA GmbH & Co., Hamburg, Germany), respectively. The measurements were recorded to the nearest 0.1 kg and 0.1 cm, respectively. The weight status of the children was determined based on the World Health Organization (WHO) growth reference for children aged 5–19 years old. Body mass index-for-age Z-score (BAZ) was determined using the WHO AnthroPlus version 1.0.3 (World Health Organisation, Geneva, Switzerland) software.

### Statistical analyses

Statistical analysis was conducted using Statistical Package for the Social Sciences (SPSS) version 22.0 (IBM SPSS Statistics 2014). Data were entered, cleaned and checked for any missing data before data analyses. Considering that the sample size was less than 100, each variable was tested for normality using the Shapiro-Wilk test. Categorical data were presented as number and percentage, while continuous data were presented as mean and standard deviation (SD). The comparisons between the IG and CG across socio-demographic data were determined using chi-square test for sex and household income (categorical data), and using t-test for age and household income (continuous data). The baseline differences between the IG and CG across scores of KAP toward whole grains were determined using independent t-tests. The present study applied a complete case analysis where missing data in the proposed model were dropped from the analysis. This analysis was more efficient than intention-to-treat analysis for a non-longitudinal quasi-experimental pre-post study, as the intention-to-treat approach often included all randomized subjects in the groups to which they were randomly assigned, regardless of their adherence to the entry criteria, the treatment they actually received, subsequent withdrawal from treatment or deviation from the protocol [26]. ANCOVA was performed to determine the effect of the GReat-Child Trial™ over the entire follow-up period, including during the 6 months post-intervention follow-up. Two models were examined, including (1) within-group differences and (2) between-groups differences. For within-group differences, the entire data was split into IG and CG, repeated measures ANCOVA within group analysis was applied followed by pairwise comparison with confidence interval adjustment. For between-groups differences, adjusted mean using repeated measures ANCOVA between group analyses was applied followed by pairwise comparison. All the models were adjusted for covariates, including all observed pre-treatment variables thought to have some connection to the outcome, all known risk factors for the outcome and all direct causes of the treatment or the outcome [27]. Hence, household income and baseline variables were considered as covariates in each model in order to prevent bias. Model assumptions, including normality of the residuals, homogeneity of variance, compound symmetry and homogeneity of regression, were verified. *P*-values of less than 0.05 and 0.001 for a two-sided test were considered statistically significant, and were marked as ^*^ and ^***^, respectively.

## Results

### Attendance rate for nutrition education classes and acceptance level of GReat-child trial™ among the children in the intervention group

**Fig. 1:**
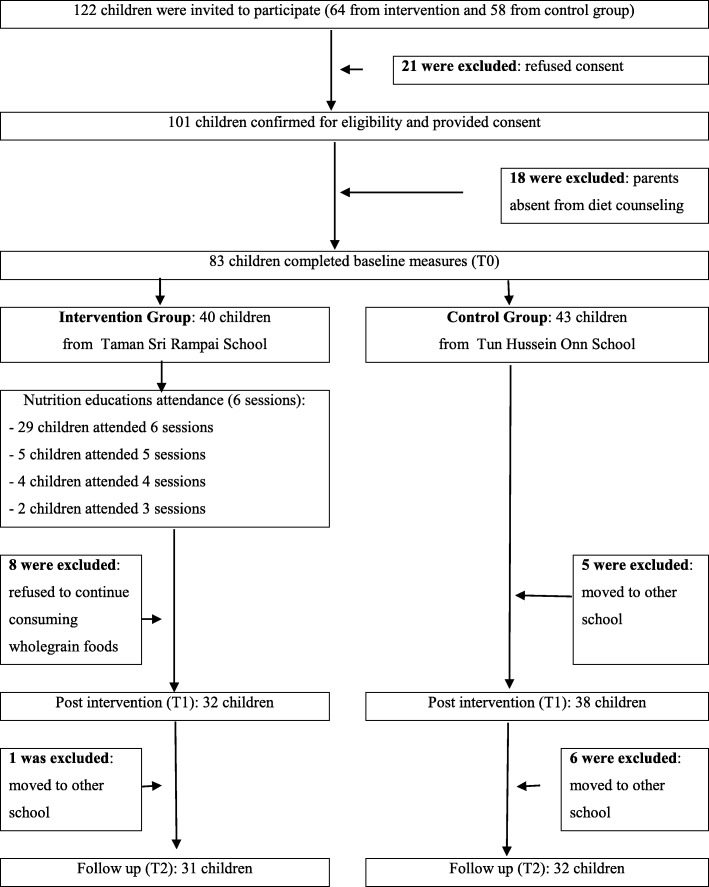
Flow of participants through the GReat-Child Trial™

Figure 1 describes the flow of participants through the trial. A total of 122 potential children who fulfilled the inclusion criteria were invited to participate in the GReat-Child Trial™ (64 from IG; 58 from CG), whereby 101 consented and agreed to take part, resulting in a response rate of 82.7%. Of the 101 children who agreed to take part, only 83 were included at baseline enrolment (40 IG; 43 CG). Eighteen IG children were excluded from the study due to the absence of their parents at the individual diet counselling session. Overall, attendance at the six 30-min nutrition education classes was higher at the first few compared to the last few classes throughout the period of the intervention. Out of the 40 children in IG, a total of 29 children (72.5%) completed all six 30-min nutrition education classes, five children (12.5%) missed one class, four children (10%) missed two classes and another two children (5%) missed three classes.

Table 2 showed the acceptance of the GReat-Child Trial™. Evaluation measures were collected at the end of the intervention. A total of 40 children in IG completed the feedback questionnaire. Overall, a majority of the children (*n* = 25; 62.5%) answered “strongly agree” to all questions in the questionnaire, this indicated a positive response to the acceptance of GReat-Child Trial™. The first five questions which assessed the perception of how much knowledge they have gained, obtained positive responses from the children. In relation to the intervention components, demonstration, explanation in nutrition education classes and the arrangement for the GReat-Child Trial™, the children were also provided positive responses.

**Table 2:** Acceptability of the GReat-Child Trial™ among all children from intervention group (*n* = 40)

Evaluation questions	Total number of children; n (%)			
	Strongly agree	Agree	Disagree	Strongly disagree
I understood the overall learning objectives in the GReat-Child Trial.	28 (70.0)	8 (20.0)	4 (10.0)	–
Through the GReat-Child Trial, my knowledge in whole grain and healthy balanced diet was enhanced.	28 (70.0)	10 (25.0)	2 (5.0)	–
Through GReat-Child Trial, my skill in identifying whole grain foods was enhanced.	26 (65.0)	12 (30.0)	2 (5.0)	–
Through GReat-Child Trial, my ability in whole grain’s knowledge and skill sharing with my family and friends was enhanced.	25 (62.5)	14 (35.0)	1 (2.5)	–
I understood all the contents in the nutrition education classes.	25 (62.5)	14 (35.0)	1 (2.5)	–
I understood the examples of whole grain foods which have been demonstrated in the nutrition education classes.	26 (65.0)	14(35.0)	–	–
I was satisfied with the facts and examples explained in the nutrition education classes.	27 (67.5)	13 (32.5)	–	–
I was involved actively in the nutrition education sessions.	25 (62.5)	14 (35.0)	1 (2.5)	–
I was given ample opportunity to clarify my questions.	30 (75.0)	10 (25.0)	–	–
I was satisfied with the GReat-Child Trial’s schedule arrangement.	28 (70.0)	10 (25.0)	1 (2.5)	1 (2.5)
I was satisfied with the GReat-Child Trial’s venue arrangement.	29 (72.5)	9 (22.5)	2 (5.0)	–
I was satisfied with the GReat-Child Trial’s module.	26 (65.0)	12 (30.0)	2 (5.0)	–
I was satisfied with the whole grain foods which were served during recess time.	26 (65.0)	6 (15.0)	7 (17.5)	1 (2.5)

By the end of the intervention, a total of 20 children (24.1%) had discontinued from the GReat-Child Trial™ either due to family relocation (*n* = 12; 14.5%) or refusal to continue the consumption of whole grain foods (*n* = 8; 9.6%) despite knowing the benefits of whole grain from the nutrition education classes. This group of children named two predominant barriers toward whole grain intake, namely the texture and the taste of the whole grain foods.

### Baseline socio-demographic, physical characteristics and scores of knowledge, attitudes and practices toward whole grain among children who successfully completed the entire trial

Baseline socio-demographic and physical characteristics, and scores of knowledge, attitudes and practices (KAP) toward whole grains consumption of the children who successfully followed through the entire GReat-Child Trial™ program are presented in Table 3. For comparison, Table 5 in Appendix shows the same results for all the children who had enrolled in study starting from baseline recruitment but had dropped out along the way during the intervention. Among the children who successfully followed through the entire intervention (*n* = 63), more than 50% of respondents were male and mean aged 10.5 years old. The majority of children were from familes with medium household income, based on the classification by the Malaysian Economic Planning Unit (2010). At baseline, mean BAZ of children in both the IG and CG were within the obese category. Knowledge score of IG was 6.7, attitudes score 45.4, and practice score 16.7. In CG, knowledge, attitudes and practice scores were 7.0, 45.4, and 16.8, respectively. There were no significant differences between the IG and CG across demographic characteristics, physical characteristics and scores of KAP toward whole grains at baseline. Similar results were shown among the whole group of children including the drop-outs (*n* = 83). Comparing the results in Table 3 and Table 5 in Appendix, we conclude that the socio-demographic and physical characteristics, as well as KAP scores were similar among the intention-to-treat group, as well as the whole group including drop-outs.

**Table 3:** Baseline socio-demography, physical characteristics and knowledge, attitudes and practices’ scores toward whole grains among children who successfully completed the entire trial (n = 63)

	Total (n = 63)	Intervention (*n* = 31)	Control (*n* = 32)	*p-*value
Age; mean ± SD	10.6 ± 0.6	10.7 ± 0.6	10.6 ± 0.6	0.882
Sex				0.262
Boys; n (%)	33 (52.4)	18 (58.1)	15 (46.9)	
Girls; n (%)	30 (47.6)	13 (41.9)	17 (53.1)	
Household income; mean ± SD	4052.4 ± 1873.8	4506.5 ± 2384.9	3612.5 ± 1054.6	0.058
Low (< RM2300); n (%)	6 (9.5)	3(9.7)	3 (9.4)	0.452
Medium (RM2300-RM5599); n (%)	50 (79.4)	23 (74.2)	27 (84.4)	
High (≥RM5600); n (%)	7 (11.1)	5 (16.1)	2 (6.2)	
Physical characteristics				
Weight (kg); mean ± SD	47.8 ± 13.0	50.4 ± 14.9	45.2 ± 10.4	0.486
Height (cm); mean ± SD	139.8 ± 7.5	142.1 ± 8.2	137.5 ± 6.2	0.093
BMI-for-age z-score; mean ± SD	2.2 ± 0.9	2.3 ± 1.0	2.1 ± 0.8	0.324
Knowledge, attitudes and practices’ scores toward whole grain				
Knowledge; mean ± SD	6.9 ± 2.0	6.7 ± 1.9	7.0 ± 2.1	0.610
Attitudes; mean ± SD	45.4 ± 5.6	45.4 ± 5.5	45.4 ± 5.8	0.993
Practices; mean ± SD	16.7 ± 2.2	16.7 ± 2.0	16.8 ± 2.4	0.852

Age, weight, height, BMI-for-age z-score, household income (in mean ± SD), knowledge, attitudes and practices toward whole grains variables were tested using Independent t-test; Sex and household income (in categories) variables were tested using Chi-square test; SD: standard deviation; Full marks for knowledge domain = 15 marks; Full marks for attitudes domain = 75 marks; Full marks for practice domain = 50 marks

### The intervention effects: between-group differences

**Fig. 2:**
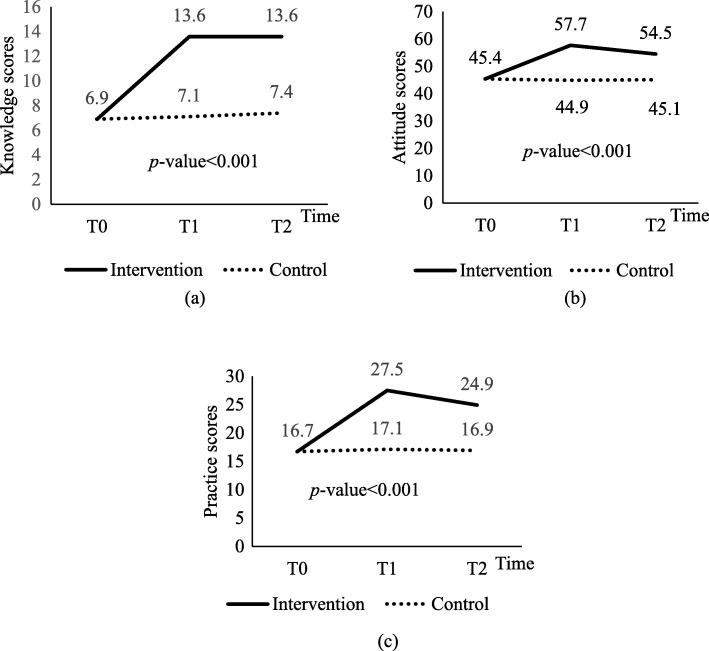
Between-group comparison of scores of knowledge, attitudes and practices toward whole grains over 9 months (*n* = 63)

Comparison between the IG and CG in scores of KAP toward whole grains over 9 months are demonstrated in Fig. 2. Overall, IG substantially increased KAP toward whole grains. IG showed significantly higher scores of knowledge (weighted difference: 4.23; 95% CI: 3.82, 4.64; *p* < 0.001), attitudes (weighted difference: 7.39; 95% CI: 6.36, 8.42; *p* < 0.001) and practices (weighted difference: 6.13; 95% CI: 4.49, 7.77; *p* < 0.001) throughtout the study period as compared to CG.

### The intervention effects: within-group differences

Ninth month changes in scores of KAP within-group are presented in Table 4. IG had significantly higher score of knowledge (weighted difference: 6.77; 95% CI: 6.45, 7.10; *p* < 0.001), attitudes (weighted difference: 12.45; 95% CI: 11.41, 13.49; *p* < 0.001), and practices (weighted difference: 10.58; 95% CI: 8.93, 12.24; *p* < 0.001) at T1 compared to baseline (T0). However, the effects were diminished in the following 6 months follow-up. IG children showed a tendency toward higher scores in knowledge at T1 and T2. However, there was no significant change in knowledge scores in the IG at T1 and T2. The same group of children showed significantly lower scores in attitudes (weighted difference: -3.29; 95% CI: − 4.37, − 2.21; *p* < 0.001), and practices (weighted difference: -2.55; 95% CI: − 4.59, − 0.50; *p* = 0.016) at T2 compared to T1. Nonetheless, IG obtained significantly higher scores of knowledge (weighted difference: 6.84; 95% CI: 6.53, 7.15; *p* < 0.001), attitudes (weighted difference: 9.16; 95% CI: 8.08, 10.24; *p* < 0.001), and practices (weighted difference: 8.03; 95% CI: 5.34, 10.73; *p* < 0.001) at T2 compared to T0.

**Table 4:** Changes in within-group scores of knowledge, attitudes and practices toward whole grains (n = 63)

	Comparison	Intervention group		Control group	
		Mean (95% CI)	*p*-value	Mean (95% CI)	*p*-value
Knowledge score	T1-T0	6.8 (6.5,7.1)	< 0.001***	0.1 (−0.3,0.5)	0.508
	T2-T0	6.8 (6.5,7.2)	< 0.001***	0.4 (−0.2,1.0)	0.222
	T2-T1	0.1 (− 0.2,0.3)	0.587	0.3 (− 0.2,0.7)	0.277
Attitudes score	T1-T0	12.5 (11.4,13.5)	< 0.001***	−0.7 (−2.0,0.6)	0.286
	T2-T0	9.2 (8.1,10.2)	< 0.001***	−0.4 (−1.5,0.6)	0.407
	T2-T1	−3.3 (−4.4,-2.2)	< 0.001***	0.3 (−0.4,0.9)	0.412
Practice score	T1-T0	10.6 (8.9,12.2)	< 0.001***	0.5 (−1.0,2.0)	0.505
	T2-T0	8.0 (5.3,10.7)	< 0.001***	0.3 (−1.2,1.8)	0.664
	T2-T1	−2.6 (−4.6,-0.5)	0.016*	−0.2 (−0.5,0.2)	0.259

T0 - Baseline; T1 – post-intervention (thirteenth week); T2 – follow-up (ninth month); ^***^Statistically significant at *p-*value < 0.001; ^*^Statistically significant at *p*-value < 0.05; Repeated measures ANCOVA within group analysis was applied followed by pairwise comparison with confidence interval adjustment; Household income dan baseline variables were controlled by using repeated measures

ANCOVA

On the other hand, children in CG did not show any significant changes in all of the scores at T0 and T1, but a tendency toward lower score in attitudes, and a tendency toward higher scores in knowledge and practices were seen over the 13 weeks. Similar outcomes were reported from T0 toT2, where children in the CG showed a tendency toward lower score in attitudes, and a tendency toward higher scores in knowledge and practices at T2 compared to T0. While these tendencies were found, none of the changes in the CG chidren were significant.

## Discussion

To the best of our knowledge, this is the first reported quasi-experimental multi-component intervention utilizing the SCT as a strategy to improve knowledge, attitudes and practices (KAP) of whole grains consumption among Malaysian children. Furthermore, this study was the first trial that has focused on whole grain to manage childhood obesity in Malaysia [6]. This intervention provides novel data and reveals that the nutrition education intervention embedded in the availability of whole grain foods environment and individual dietary counseling based on SCT has led to significant improvements in KAP of self-reported whole grain consumption among children. Given that greater consumption of whole grain has been shown to improve weight management among children [6], these positive findings from the trial have the potential to impact the long-term management of childhood obesity in Malaysia by improving their whole grain intake.

We believe that the observed changes in the KAP outcomes can be attributed to the feasibility and acceptability of the intervention delivered. A crucial stage in an intervention’s development and optimization is to assess elements associated with the feasibility and acceptability of the intervention [28]. Acceptability of the intervention, effect sizes, participants’ satisfaction with the intervention, as well as attrition and adherence rates are all important components of a feasibility trial that provide insights into the effectiveness, structure and potential improvement of an intervention [29]. Feasibility and positively accepted trials are an important precursor to the implementation of behavior change interventions [30]. Studies that have evaluated the feasibility and acceptability of whole grain-related nutrition education interventions among children are scarce. The overall study attrition rate was considerably higher compared to a similar whole grain intervention in the United States; with a total of 24.1% of children in the current study failing to complete the intervention, compared to a zero drop-out rate from the whole grain intervention in the United States [19]. This could be because whole grain foods are more accepted as a cultural norm in the United States, whereas they are not so commonly consumed and less culturally accepted in Malaysia [13]. It is noteworthy that the addition of nutrition education and daily whole grain foods delivery components within the GReat-Child intervention did not, in this instance, marginalize the IG children’s retention.

Compliance to the nutrition education classes was good, with children completing 98.4% of the scheduled sessions (after accounting for dropouts). It might be due to the arrangement of nutrition education classes, where the IG received the nutrition education classes on Wednesday afternoons at 1.00 p.m., right after finishing their regular school lessons, and before they began co-curricular activities, which is implemented at 1.30 p.m. based on guidelines from the Malaysian Ministry of Education. This arrangement was made so that the delivery of the nutrition education would not interfere with regular school and co-curricular activities, as well as to minimize any inconvenience to all parties concerned [22].

Positive response for questions assessing the GReat-Child Trial’s module, demonstration, explanations in nutrition education classes and the whole grain foods delivery arrangement; supported the positive acceptability of the GReat-Child Trial™. This is because the selected whole grain foods, such as whole grain ready-to-eat cereals, whole grain bread and whole grain biscuits, were provided to the children to substitute food that the children would normally eat during recess time. This arrangement was made so that 100% of the whole grain foods distributed would be eaten by the children under the researcher’s supervision. For any children who were absent on a particular day, the whole grain foods were distributed to them the next day. The absent child would bring the extra whole grain foods home for consumption over the weekend. To ensure the children followed the instructions given, parents were reminded via phone calls or via text messages on that particular weekend. The whole grain foods that were provided as part of their regular ration, which was not consumed during recess time on school days due to their absence, were taken as breakfast during the weekend and monitored by their parents. The selected whole grain foods were nutrient-dense, convenient, and easy to prepare [22]. However, eight children from the IG were unable to accept the whole-grain biscuits, whole-grain bread and whole-grain ready-to-eat cereals. The limited usage of self-monitoring and self-efficacy factors are important to highlight, as previous research has shown that optimal results in behavior change interventions arise when these factors were utilized effectively [31]. However, taken together, all the components of GReat-Child Trial™ did not detract from the IG children’s adherence, attendance or enjoyment of the intervention.

Knowledge of whole grain improved in both the IG and CG. This is in concordance with the findings of a meta-analyses, where quality curriculum interventions largely-based on behavioral or SCT were found to be capable of achieving improvements in primary children’s nutritional knowledge [32]. Results from the present study with regards to knowledge are consistent with a whole grain intervention from the United States [19]. Establishing good whole grains-related knowledge is an essential first step to improving children’s self-efficacy to choose whole grain foods in several situations [19]. CG showed a slight improvement in the knowledge domain from baseline to follow-up, which may be a function of repeat administration of the questionnaire [33], but these differences were not significant. Additionally, IG performed significantly better than CG.

We are not aware of any other experimental trial measuring the attitudes toward whole grain consumption among children. However, the attitudes outcome of the present study is consistent with findings from previous school-based nutrition interventions which utilized SCT, that aimed to improve overall dietary intake, but were not specific in whole grain, among children in Lebanon [34] and Malaysia [35]. The differences in attitudes-evaluation scores at pre- and post-intervention highlight a number of areas of interest. The positive outcomes in the present study could be explained by the adoption of a theory-driven approach in our intervention development, mainly the SCT [34]. A systematic review demonstrated that evidence-base constructs are effective in changing dietary knowledge and attitudes [36]. Observational learning was achieved through the whole grain foods preparation during recess time, and promotion of behavioral capability and self-efficacy among children through quizzes and activities during nutrition education classes, built the confidence of children to identify healthy whole grain meal and snack choices. In addition, the use of a multi-component approach that included nutrition education classes combined with provision of whole grain foods delivery during recess time could have contributed to the positive improvement in their attitudes scores.

Overall, a wide range of whole grain foods in the present study was considered acceptable by the children. The IG maintained a significantly higher score of practices toward whole grain consumption throughout the trial period. There was a great deal of evidence that showed that repeated exposure to foods that children spontaneously elect not to consume can increase subsequent consumption in long-term [37]. However, repeated taste exposure in the absence of any other motivational education classes will not effectively ensure that the children would continuously consume those particular foods [38]. In the present trial, the significant increase of practice scores in the IG is very likely attributed to the three components addressing the personal, behavior and environmental factors based on the SCT in our 12-week program. The Great-Child Trial™ takes into consideration personal factors, such as outcome expectation, self-efficacy and self-regulation; behavior factors, including the usual food choice and behavioral capability; and environmental factors, such as accessibility and availability of whole grain foods [22]. The SCT foundation successfully assists the parents and children in raising their awareness of whole grain consumption and healthy balanced diet, as well as motivates the children and parents to change their dietary intake.

Although the outcomes of the GReat-Child Trial™ showed statistically significant results on KAP toward whole grains consumption in 12 weeks, the effects were diminished in the following 6 months. This implies that consumption adherence is weak. The higher initial levels of whole grain foods consumption in T1 may then be the result of a novelty effect of the introduction of whole grain. Indeed, this finding is consistent with previous research that has also shown a decline over time in consumption of fruit introduced at school lunch among children [38]. A number of reasons had been suggested as to why Malaysian children choose refined grains options as opposed to whole grain, may be due to texture and taste of whole grain foods [13, 17]. To maximize the effectiveness of the trial, a longer intervention period with additional strategies to assist in maintaining whole grains consumption in the long-term may be needed, as it seems plausible that an intervention with whole grains for a longer time period would be likely to increase acceptability of such products. Future studies assessing the importance of time length of intervention and proportion of whole grain necessary to effect a long-term dietary change therefore seem pertinent [39]. It should be noted, as a practical consideration, that whole grain products are comparatively more expensive and has lower accessibility in Malaysia [13]. White rice is relatively cheap and widely available in Malaysia, whereas brown rice is more expensive and available only in hypermarkets [13]. Perhaps public health strategies such as those employed by Singapore could be considered for Malaysia; for example, low-price vendors are subsided for offering healthier choices, including for whole grain foods [39].

Although the findings of this study are promising, it has several limitations that need to be considered. One such limitation, assessment of KAP on whole grains consumption in children is particularly challenging, as children might not have told the truth, especially when answering questions on attitudes and practices as it could, introduce social desirability bias. To minimize this limitation, the researcher had re-assured the children of their anonymity and confidentially of individual results. Another recognized limitation of this study is that the non-blinded method may overestimate intervention effects compared with blinded method. In situations when the subjects are assigned to interventions like dietary, educational or behavior change, it is not feasible to blind the participants [40]. To minimize this limitation, the researcher had blinded the outcome assessors.

Despite these limitations, the present trial has several strengths worth noting. The GReat-Child Trial™ represented a novel approach to synthesize and profile the SCT to examine the effectiveness of intervention in improving KAP toward whole grains. Household income was controlled as a covariate, which had been previously shown to be susceptible to whole grain consumption in Malaysian children [13].

## Conclusion

On the whole, the present study demonstrated that an intervention that incorporates whole grain foods and healthy balanced diet, which utilized the SCT, may significantly improve KAP of whole grains consumption among children who were overweight and obese. Findings from the present study also demonstrated that the availability of whole grain foods at school can increase its consumption among children. The successful outcome of this study is relevant to the School Breakfast Programme being planned by the Ministry of Education Malaysia; and policies regarding the inclusion of whole grains in the preparation of foods served at the school canteen will be implemented. Future collaborations may be conducted between the industry, government and universities to scale up the intervention. We anticipate that the GReat-Child Trial™ intervention could be executed at schools throughout Malaysia in order to improve whole grains consumption among Malaysian children for obesity prevention.

### Supplementary information

**Additional file 1.** Questionnaire of knowledge, attitudes and practices toward whole grains consumption among Malaysian schoolchildren.

## Data Availability

The datasets used and/ or analyzed during the current study are available from the corresponding author on reasonable request.

## Appendix

**Table 5:** Baseline socio-demography, physical characteristics and knowledge, attitudes and practice scores toward whole grains among children who enrolled in baseline recruitment (*n* = 83)

	Total (*n* = 83)	Intervention (*n* = 40)	Control (*n* = 43)	*p-*value
Age; mean ± SD	10.6 ± 0.6	10.6 ± 0.6	10.6 ± 0.6	0.882
Sex				0.219
Boys; n (%)	44 (53.0)	24 (60.0)	20 (46.5)	
Girls; n (%)	39 (47.0)	16 (40.0)	23 (53.5)	
Household income; mean ± SD	3977.1 ± 1759.6	4467.5 ± 2229.5	3520.9 ± 993.7	0.159
Low (< RM2300); n (%)	7 (8.4)	4 (10.0)	3 (7.0)	0.134
Medium (RM2300-RM5599); n (%)	67 (80.7)	29 (72.5)	38 (88.4)	
High (≥RM5600); n (%)	9 (10.9)	7 (17.5)	2 (4.6)	
Physical characteristics				
Weight (kg); mean ± SD	47.0 ± 11.9	50.1 ± 13.5	44.1 ± 9.5	0.754
Height (cm); mean ± SD	139.1 ± 7.2	141.3 ± 7.9	137.0 ± 5.9	0.533
BMI-for-age z-score; mean ± SD	2.3 ± 0.9	2.5 ± 1.0	2.1 ± 0.8	0.106
Knowledge, attitudes and practices’ scores toward whole grain				
Knowledge; mean ± SD	6.8 ± 1.8	7.1 ± 1.8	6.4 ± 1.8	0.294
Attitudes; mean ± SD	47.1 ± 6.5	45.7 ± 6.7	48.5 ± 6.1	0.538
Practices; mean ± SD	16.7 ± 2.2	16.7 ± 1.9	16.8 ± 2.4	0.815

Age, weight, height, BMI-for-age z-score, household income (in mean ± SD), knowledge, attitudes and practices toward whole grains variables were tested using Independent t-test; Sex and household income (in categories) variables were tested using Chi-square test; SD: standard deviation; Full marks for knowledge domain = 15 marks; Full marks for attitudes domain = 75 marks; Full marks for practice domain = 50 marks

## Abbreviations

BAZ: Body mass index-for-age Z-score
CG: Control group
IG: Intervention group
KAP: Knowledge, attitudes and practices
SCT: Social Cognitive Theory
SD: Standard deviation
SPSS: Statistical Package for the Social Sciences
T0: Baseline
T1: First post-intervention (at week 13)
T2: Second post-intervention (at 9th month follow-up)
