# Supplementary material for: GReat-Child Trial™ based on social cognitive theory improved knowledge, attitudes and practices toward whole grains among Malaysian overweight and obese children
Source: BMC Public Health. 2019 Nov 27;19:1574. doi: 10.1186/s12889-019-7888-5 (PMC6881981; doi:10.1186/s12889-019-7888-5)
Supplement: Supplementary file 1 — Additional file 1. Questionnaire of knowledge, attitudes and practices toward whole grains consumption among Malaysian schoolchildren. [file 12889_2019_7888_MOESM1_ESM.docx]

**SOAL SELIDIK PENGETAHUAN, SIKAP DAN AMALAN KANAK- TERHADAP BIJIRIN PENUH**

**Arahan: Sila tandakan ( / ) pilihan yang tepat pada ruangan yang disediakan. Pilih SATU jawapan sahaja untuk setiap soalan**

| **NO** | **PENGETAHUAN TENTANG BIJIRIN PENUH** |
| --- | --- |
| K01 | Piramid makanan berbentuk _____________.  Segiempat  Segitiga  Tidak pasti |
| K02 | Aras _____ piramid makanan mengandungi kumpulan makanan yang kaya dengan karbohidrat kompleks.  Pertama  Kedua  Tidak pasti |
| K03 | Mengikut piramid makanan, kumpulan makanan yang boleh dimakan dengan kuantiti yang secukupnya ialah:  Sayur dan buah-buahan  Bijirin penuh dan ubi  Tidak pasti |
| K04 | Makanan yang mengandungi _________________ merupakan sumber tenaga utama yang diperlukan oleh tubuh badan kita.  Vitamin  Karbohidrat kompleks  Tidak pasti |
| K05 | Antara yang berikut, makanan yang merupakan sumber karbohidrat kompleks ialah.  Ikan  Bijirin penuh  Tidak pasti |
| K06 | Apakah itu bijirin penuh?  Bijirin penuh merupakan bahagian daun yang boleh dimakan  Bijirin penuh merupakan bahagian biji benih yang boleh dimakan  Tidak pasti |
| K07 | Antara yang berikut, makanan yang merupakan bijirin penuh ialah  Nasi putih  Jagung  Tidak pasti |
| K08 | _______________ merupakan bijirin penuh.  Oat  Ubi keledek  Tidak pasti |
| K09 | Bijirin penuh sedia dimakan kaya dengan _____________.  Air  Asid folik (vitamin B9)  Tidak pasti |
| K10 | Makanan berbijirin penuh kaya dengan _____________.  Zink  Lemak  Tidak pasti |
| K11 | Bijirin penuh mengandungi __________ yang tinggi.  Garam  Serat  Tidak pasti |
| K12 | Pengambilan bijirin penuh sedia dimakan pada waktu rehat sebagai sarapan pagi memberikan rasa kenyang yang lebih lama.  Ya  Tidak  Tidak pasti |
| K13 | Apakah fungsi utama serat yang terdapat dalam bijirin penuh sedia dimakan?  Membantu dalam pembinaan tisu badan  Membantu dalam pencernaan dan penghadaman  Tidak pasti |
| K14 | Bijirin penuh membantu dalam ______________.  Peningkatan berat badan  Penurunan berat badan  Tidak pasti |
| K15 | Mengapa pengambilan bijirin penuh sedia dimakan sebagai sarapan pagi membantu dalam menguatkan tulang?  Kerana pengambilan bijirin penuh sedia dimakan meningkatkan  pengambilan lemak  Kerana pengambilan bijirin penuh sedia dimakan meningkatkan  pengambilan kalsium  Tidak pasti |

**Arahan: Sila tandakan ( / ) pada ruangan yang disediakan mengikut pandangan anda terhadap setiap pernyataan di bawah.**

| **NO** | **SIKAP TERHADAP BIJIRIN PENUH** | **Sangat Setuju**  **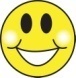** | **Setuju**  **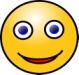** | **Tidak Pasti**  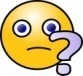 | **Tidak Setuju**  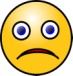 | **Sangat Tidak Setuju**  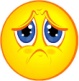 |
| --- | --- | --- | --- | --- | --- | --- |
| A01 | Saya tidak suka mengambil bijirin penuh sedia dimakan sebagai sarapan pagi kerana ia menyebabkan saya cepat merasa kenyang dan tidak dapat menikmati ayam goreng pada waktu tengahari. |  |  |  |  |  |
| A02 | Saya jarang mengambil bijirin penuh sedia dimakan kerana ia sukar didapati di tempat tinggal saya dan saya malas untuk mendapatkannya di tempat lain. |  |  |  |  |  |
| A03 | Saya akan memilih biskut berkrim manis jika terdapat pilihan antara biskut bergandum penuh dan biskut berkrim manis. |  |  |  |  |  |
| A04 | Saya tidak suka mengambil bijirin penuh sedia dimakan dengan susu rendah lemak kerana rasanya tidak sedap. |  |  |  |  |  |
| A05 | Saya rajin membaca untuk mengenalpasti fungsi bijirin penuh. |  |  |  |  |  |
| A06 | Saya cuba meluangkan masa melayari laman web yang berkaitan dengan bijirin penuh untuk memahami kebaikan bijirin penuh. |  |  |  |  |  |
| A07 | Saya cuba meluangkan masa membaca buku berkaitan dengan bijirin penuh untuk mencari sumber yang kaya dengan bijirin penuh. |  |  |  |  |  |
|  |  | **Sangat Setuju**  **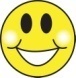** | **Setuju**  **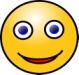** | **Tidak Pasti**  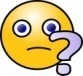 | **Tidak Setuju**  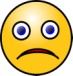 | **Sangat Tidak Setuju**  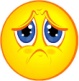 |
| A08 | Saya akan membeli bijirin penuh sedia dimakan sebagai snek pagi pada waktu rehat jika ia dijual di kantin sekolah. |  |  |  |  |  |
| A09 | Saya menghabiskan makanan berbijirin penuh walaupun saya tidak suka makanan tersebut. |  |  |  |  |  |
| A10 | Saya tidak risau sama ada memilih makanan berbijirin penuh atau tidak kerana saya masih sihat. |  |  |  |  |  |
| A11 | Saya cuba bertanya kepada guru atau ibu bapa jika terdapat sebarang soalan tentang bijirin penuh. |  |  |  |  |  |
| A12 | Saya akan memilih bijirin penuh sedia dimakan sebagai makanan sarapan pagi jika terdapat pilihan antara bijirin penuh sedia dimakan dan nasi lemak. |  |  |  |  |  |
| A13 | Saya berminat mengambil makanan berbijirin penuh jika mengetahui kebaikan pengambilan bijirin penuh. |  |  |  |  |  |
| A14 | Saya berpendapat bahawa makanan berbijirin penuh mempunyai nutrien dan khasiat yang lebih banyak berbanding dengan makanan bukan berbijirin penuh seperti roti putih dan nasi lemak. |  |  |  |  |  |
| A15 | Saya akan memilih roti putih jika terdapat pilihan antara roti gandum penuh dan roti putih. |  |  |  |  |  |

**Arahan: Sila tandakan ( / ) pada ruangan yang disediakan mengikut kekerapan anda dalam pengambilan makanan berbijirin penuh mengikut petunjuk dibawah.**

Selalu - 4-6 hari dalam seminggu

Kadang-kadang- 14 hari dalam sebulan

Jarang - Tidak termasuk dalam kategori “kadang-kadang” dan “selalu”

| **NO** | **AMALAN TERHADAP BIJIRIN PENUH** | **Tidak Pernah** | **Jarang** | **Kadang-kadang** | **Selalu** | **Setiap Hari** |
| --- | --- | --- | --- | --- | --- | --- |
| P01 | Saya mengambil bijirin penuh sedia dimakan dengan susu rendah lemak sebagai snek. |  |  |  |  |  |
| P02 | Saya mengambil bijirin penuh sedia dimakan tanpa susu sebagai snek. |  |  |  |  |  |
| P03 | Saya mengambil jagung sebagai snek. |  |  |  |  |  |
| P04 | Saya mengambil biskut bergandum penuh sebagai snek. |  |  |  |  |  |
| P05 | Saya mengambil oat semasa saya rasa lapar. |  |  |  |  |  |
| P06 | Saya mengambil air barli sebagai minuman. |  |  |  |  |  |
| P07 | Saya mengambil nasi perang sebagai hidangan utama. |  |  |  |  |  |
| P08 | Saya mengambil bijirin penuh sedia dimakan dengan susu rendah lemak untuk sarapan pagi. |  |  |  |  |  |
| P09 | Saya mengambil bijirin penuh sedia dimakan dengan minuman coklat sebagai makanan sarapan pagi. |  |  |  |  |  |
| P10 | Saya mengambil roti berbijirin penuh sebagai makanan sarapan pagi. |  |  |  |  |  |
